# A Subtle Profile With a Significant Impact: Language and Communication Difficulties for Autistic Females Without Intellectual Disability

**DOI:** 10.3389/fpsyg.2021.621742

**Published:** 2021-08-09

**Authors:** Alexandra Sturrock, Catherine Adams, Jenny Freed

**Affiliations:** Department of Human Communication Hearing and Development, The University of Manchester, Manchester, United Kingdom

**Keywords:** autism, language and communication, sex/gender differences, social impact, emotional impact, functional impact

## Abstract

The presentation of autism in females is poorly understood, which is thought to contribute to missed or later- age diagnosis, especially for those without intellectual disability. Dedicated research into social and behavioral differences has indicated a specific female phenotype of autism. However, less has been done to explore language and communication profiles, despite known sex/gender differences in typically developing populations. This article provides a synthesis of recent work from this small but emerging field. It focuses on a series of four preliminary and explorative studies conducted by the authors and embeds this within the wider literature. Findings suggest a specific profile of language and communication strengths and weaknesses for autistic females without intellectual disability (compared to autistic males and typically developing females). Furthermore, despite the relatively subtle presentation of difficulties (compared to autistic males), the impact on functionality, social inter-relations and emotional well-being, appears to be equitable and significant. The discussion highlights the need for further empirical research and proposes areas for investigation. Implications for clinical practice include the need for better recognition, testing and provision of interventions dedicated to the language and communication difficulties for autistic females. This has relevance for diagnostic, mental health and speech and language therapy services.

## Introduction

Sex/gender^1^ differences in language and communication profiles for typically developing individuals are well documented in the literature. Females demonstrate earlier acquisition of first words (Bleses et al., 2008), better and earlier integration of language with gesture (Eriksson et al., 2012), earlier examples of social-emotional vocabulary (e.g., “like,” “please”), and use of more complex linguistic forms during spontaneous speech (Bouchard et al., 2009). They also use language and communication differently from males, focusing on person-centered topics and emotions (Newman et al., 2008), and using collaborative and negotiated discourse (Ladegaard and Bleses, 2003). Importantly, this profile appears to be expected within interactions (Newman et al., 2008) and is linked to successful integration with female social groups (Tierney et al., 2016).

Sex/gender differences in autism have received growing attention in recent years, although this has focused on social and behavioral domains rather than language and communication. Currently females are diagnosed in lower numbers (1:3) than males (Loomes et al., 2017) especially in groups with higher cognitive function (1:7; Nicholas et al., 2008). This is despite autistic symptomatology existing with relative parity (2:1) in whole population samples (Giarelli et al., 2010). Clinical concerns are that females are being missed from diagnosis due to poor recognition of the autistic female phenotype (Kreiser and White, 2014). Sex/gender differences have been identified in rigid/repetitive behaviors using diagnostic measures (Van Wijngaarden-Cremer et al., 2014; Hull et al., 2017a) with males typically exhibiting increased frequency and severity compared to females. Differences in social interactions have been better identified using specific measures, avoiding the homogenizing effect of collecting data and constraining participant groups using the same diagnostic tools (Lai et al., 2015). Several studies now point toward a distinct profile of social-interaction difficulties for females compared to males, using measures of empathizing (Rieffe et al., 2021), friendship (Sedgewick et al., 2016), play-behaviors (Dean et al., 2014), and emotional reciprocity (Head et al., 2014). A review of the literature found little evidence of language and communication differences between sex/gender in autism (Hull et al., 2017a). However, data in those studies were collected using isolated measures (parental reports or basic vocabulary tasks), where difference may be under-identified for reasons discussed in this paper. Others used diagnostic measures, which may incur a homogenizing effect by constraining participants and measuring difference using the same tools (Lai et al., 2015). This current article focuses on the smaller body of work investigating subtle sex/gender difference using specific measures of language and communication, in pragmatic and above sentence-level language. Principally, it will consider four clinically driven studies from the authors’ research group; using direct assessment (Sturrock et al., 2019b), observation and report measures (Sturrock et al., 2019a), child interviews (Sturrock et al., 2021) and parental interviews (Sturrock et al.,), and synthesizes these with recent findings from the wider literature. It proposes that autistic females most likely to be missed from diagnosis (those without intellectual disability: IQ ≥ 70) have a specific profile of language and communication skills, different from both autistic males and typically developing females, and that these differences make them prone to negative social, functional and emotional sequelae. It calls for further research and proposes areas for investigation.

## Assessment of a Subtle Profile of Difficulties

While subtle language and communication differences are identified between autistic individuals (without intellectual disability) and typically developing (TD) controls (Howlin, 2003; Kelley et al., 2006), this is rarely achieved through basic structural language assessment (e.g., testing vocabulary and sentence-level grammar). Neither is basic structural language expected to differ between school-aged and above TD females and males (Newman et al., 2008). An attempt to explore sex/gender difference must therefore utilize measures with the capacity to compare subtly differing profiles.

**FIGURE 1:**
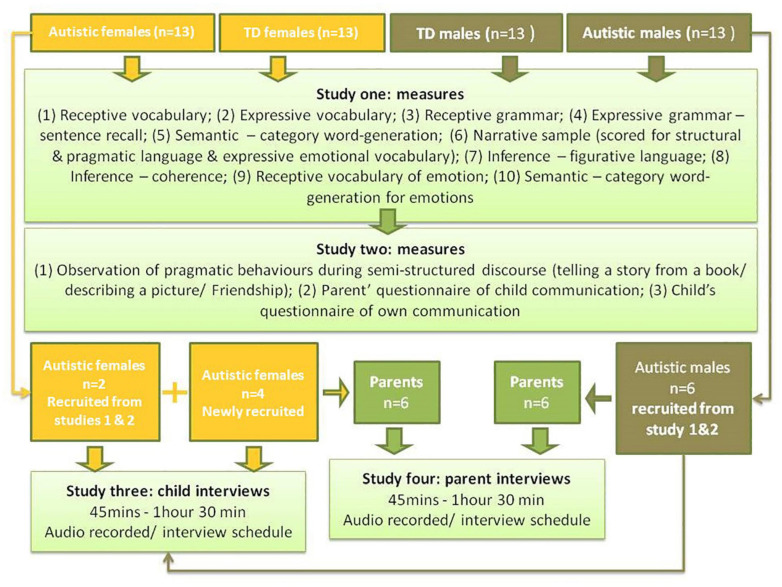
Flow diagram of participants and measures over four studies of language and communication.

Sturrock et al. (2019b) proposed a battery of direct assessments targeting language (expressive and receptive) at multiple levels (word, sentence and above sentence-level/narrative), word knowledge (semantics), inference and vocabulary of emotion. In subsequent work, the authors proposed a series of functional communication measures (Sturrock et al., 2019a) including parent and child questionnaires and observational checklists for social use of language (pragmatic skills). Details of assessment measures are found in Supplementary Appendix 1. These measures were undertaken with a cohort of 52 children without intellectual disability in a 2 (diagnosis: Autism/TD) by 2 (sex/gender: female/male) design. Children were recruited from a narrow age range (8y11m–11y6m), to minimize the effect of increasing language abilities across development. Children in middle childhood were purposefully selected, being young enough to avoid interference of secondary mental health conditions (social communication difficulties are thought to increase in secondary school for autistic girls; 6) but old enough to be post- diagnosis (likely to occur much later for autistic girls (Rutherford et al., 2016). Overall, participants had PIQ ≥ 70, and there were no statistical differences on basic vocabulary and grammar skills or autism severity between groups (see Supplementary Appendix 2). Figure 1 provides a depiction of assessment measures per child.

As predicted from the literature (Howlin, 2003; Kelley et al., 2006) no group differences were identified in receptive or expressive vocabulary or sentence-level language. However, it is possible that other measures may have provided a more discrete assessment of difference; for example, The Index of Productive Syntax (Scarborough, 1990) showed group differences in expressive sentence-level grammar when comparing spontaneous language samples of autistic children without learning disability and TDs (Eigsti et al., 2007). Similarly, subtests for following oral instruction within the CELF (Semel et al., 1987) and NEPSY (Korkman et al., 1997) assessment batteries, demonstrated problems in receptive ability (Koning and Magill-Evans, 2001; Saalasti et al., 2008) for autistic children without intellectual disability compared to controls. Sex/gender differences in these language subtests have not been explored but may have better capacity for identifying subtle variations and are worthy of investigation. Another consideration is the existence of heterogeneity amongst autistic individuals and the probable existence of a subgroup with specific grammatical language impairment (Roberts et al., 2004; Wittke et al., 2017). Similar to the non-autistic population specific language difficulties can occur in autism without other intellectual disability, the prevalence of this within autistic girls is currently unknown. In larger population studies, it would be important to isolate this group for separate consideration in analysis. The findings from the author’s series of studies focuses on the profile of autistic girls without such additional and specific grammatical difficulties, as evidenced by the children’s performance on the basic structural language tasks.

## Sex/Gender Difference in Narratives

Narrative has been used to demonstrate subtle deficits in the language and communication skills of autistic individuals without intellectual disability, even when basic structural language is in normal range. Narrative requires the individual to recall, organize and present information in a way that orients the listener to story meaning; blending cognitive and linguistic skills (Norbury et al., 2014) with an ability to interpret social cues from the listener (Volden et al., 2017). Mixed-sex/gender or male autistic groups without intellectual disability have demonstrated deficits in structural (Diehl et al., 2006; Rumpf et al., 2012; McCabe et al., 2013) and pragmatic (Capps et al., 2000; Losh and Gordon, 2014; Banney et al., 2015; Kauschke et al., 2016) features of narrative. It therefore provides scope for demonstrating differences in higher-level language and communication profiles and potentially between sex/gender.

Sturrock et al. (2019b) found autistic females and males performed similarly but behind TDs in their use of temporal connectors (“and then.”) and number and range of causal connectors (“so.”) leading to overall limitations with structural complexity and pragmatic coherence. This may potentially support the argument for subtle group differences in higher-level linguistic competency (Kelley et al., 2006; Eigsti et al., 2007; Saalasti et al., 2008). Other studies have demonstrated sex/gender differences in pragmatic elements of narrative, with autistic females generating richer character depictions and descriptions of internal states, cognition, perception and judgment (Kauschke et al., 2016; Boorse et al., 2019; Conlon et al., 2019) and overall better skills in retelling salient story elements (Conlon et al., 2019). When compared to typically developing peers, however, autistic girls experienced difficulties on these measures (Kauschke et al., 2016). Sturrock et al. (2019b) also found autistic females and males performed behind typically developing children in their use of vocabulary of emotion in narrative. These relative difficulties for autistic girls compared to TDs may put them at a functional disadvantage in terms of social integration (Dean et al., 2014) and self-advocacy (Sillar et al., 2014). The need for integrating linguistic information with social cues (Volden et al., 2017) may explain better outcomes for autistic females on pragmatic elements of narrative. This may be grounded in other noted advantages for females; in social motivation (Head et al., 2014; Sedgewick et al., 2016) and social attention (Harrop et al., 2018). It would therefore be of interest to isolate underpinning linguistic and socio-cognitive skills in narrative and investigate the influence of sex/gender on those.

## Semantic Sex/Gender Differences

Sex/gender difference in this language and communication domain are particularly poorly investigated, despite being one of the more widely recognized linguistic impairments in autism more generally (Groen et al., 2008). However, Sturrock et al. (2019b) and Goddard et al. (2014) found that autistic females performed better than autistic males using similar word-generation/fluency tasks. They also both found that autistic girls performed behind TDs on the same measures. Sturrock et al. (2019b) asked participants to name as many words as they could from four categories (animals, food, occupations and emotions) within a 60-s limit. Raw scores for “animals,” “food,” and “occupations” were amalgamated into one composite score and analyzed separately from the category “emotions.” Unlike expressive vocabulary tasks (like the TOWK), word-generation tasks require the individual to generate multiple word examples from a single category (relying on a flexible interpretation of word meaning) and does not provide visual stimulation to aid recall. These features may explain why semantic/word-generation tasks are more commonly occur in autism (Groen et al., 2008) while expressive vocabulary may be unimpaired. Secondary analysis in Sturrock et al. (2019b), study suggested that the sex/gender differences occurred within categories as well as using the composite score. Autistic boys demonstrated relatively elevated performance in the category of “animals” which observationally was associated with specialist knowledge in this area (typified by low-frequency, highly specialist exemplars; lion-mane jellyfish, stork-eyed beetle, goblin shark). The interaction between special interests and vocabulary acquisition is an area of potential future research, which might help explain elevated idiosyncratic word choices reported in autistic groups (Walenski et al., 2008). Further, differences in performance on semantic category word-generation tasks have been associated with differences in lexical organization between autistic and non-autistic groups (Gaffrey et al., 2007), highlighting the need for investigations of sex/gender differences in mechanisms of the development of semantic organization and their relationship to outcomes on these tasks.

## Sex/Gender Differences in Pragmatics: Inference and Discourse Behaviors

Inference is identified as a persistent difficulty for autistic individuals without intellectual disability (Loukusa and Moilanen, 2009), relying on core language (Tzuriel and Groman, 2017) and social-cognition skills (Martin and McDonald, 2004). Currently, there is very limited investigation into sex/gender differences in pragmatic inference. Two tasks in Sturrock et al. (2019b) provide some early insight: one interpreting meaning from figurative language (MacKay and Shaw, 2004), the other interpreting coherence within text using world knowledge (Jolliffe and Baron-Cohen, 1999). The children were asked to explain speaker’s intended meaning and demonstrate meta-awareness of a range of figurative language examples in the first task, then asked to identify missing information implied within a short story in the second. These early investigations suggested that autistic females perform better than autistic males and worse than typically developing females on tasks requiring inferential interpretation. Further investigation is of course required. However, it is in keeping with the literature that underlying skills in social awareness may put autistic females at an advantage on these tasks. These early findings suggest important differences in inference between autistic females and males, with consequent implications for diagnosis. They point to fruitful further work investigating sex/gender difference in other measures of inference, and highlight the importance of isolating the relative impact of social cognition or linguistic ability on performance.

By contrast, sex/gender differences in pragmatic behaviors during discourse have had more attention in the wider literature. Sturrock et al. (2019a) used the Pragmatic Rating Scale (PRS; Landa et al., 1992) as a measure of observable pragmatic features within semi-structured discourse (using the Autism Diagnostic Observation Schedule-Second Edition; Lord et al., 2012). Total PRS scores (Sturrock et al., 2019a) again showed autistic females performing better than autistic males but behind typically-developing females, replicating the pattern found in pragmatic (inference) tasks (Sturrock et al., 2019b). Differences were driven by performance on discourse management, communicative use of speech and language and non-verbal skills. Although specific analysis of sex/gender differences in discourse have not yet been undertaken, they will certainly have an important impact on the social experiences of autistic individuals. For example, Cola et al. (2020) found autistic females performed better than autistic males on a measure of first impressions during naturalistic conversations. The authors proposed first impressions would be based on judgments of pragmatic behaviors such as vocal prosody, gesture, facial expressivity and general awkwardness, although this was not expressly tested. Similar findings occurred during observation of video-recorded interactions in a study by Cage and Burton (2019). Better conversational reciprocity for autistic females compared to autistic males was also identified using diagnostic criteria in DSM-IV and DSM-5 (Hiller et al., 2014) and through analysis of appropriate pause markers, e.g., “um” as opposed to “uh” during speech samples (Parish-Morris et al., 2017). It has been suggested that this could be associated with females’ masking of autistic features (Parish-Morris et al., 2017), a phenomenon associated with camouflaging autistic behaviors more generally (Hull et al., 2017b). However, pragmatic language requires skills which integrate linguistic content with social context (Baird and Norbury, 2016), and as previously described autistic females’ elevated outcomes on social measures (compared to autistic males) may be due to natural differences in social attention and motivation (Head et al., 2014; Sedgewick et al., 2016; Harrop et al., 2018). Detailed discourse analysis could contribute to better understanding of subtle differences in conversational behaviors between autistic females and males and should be compared to normative data.

## Subtle Profile and Significant Impact

Overall, then, early findings suggest that autistic females will present with a subtle profile of language and communication difficulties compared to autistic males, yet they continue to demonstrate difficulties compared to typically developing females. This mirrors findings from research into social interactions (Sedgewick et al., 2016) and play behaviors (Knickmeyer et al., 2008). Their subtle presentation, compared to autistic males, may easily confound diagnosis, limiting access to appropriate services and indirectly leading to poorer functional outcomes and emotional well-being (Bargiela et al., 2016). However, it is also important to consider whether fewer language and communication difficulties as measured by direct assessment, will equate with fewer *perceived* difficulties as reported by the individual or their parent.

The limited data appear to suggest that when asked to rate language and communication difficulties autistic females and their parents perceive a similar level of deficit as autistic males and their parents (Sturrock et al., 2019a). This was shown using the CC-SR (Bishop et al., 2009), and CCC-2 (Bishop, 2003). This may indicate equal levels of perceived difficulties experienced by autistic females and males.

Although hard to interpret, similar findings were identified when autistic individuals (Holtmann et al., 2007) and their parents (Lai et al., 2011) were asked to rate their autism severity. As with the language and communication data, females and males perceived their levels of difficulty to be equally severe, despite females presenting with lower severity on more objective measures of clinical observation. It has been hypothesized that this phenomenon is related to the higher social expectations placed on females (Holtmann et al., 2007), meaning their reduced level of difficulty could be offset by an increased level of demand. It could also demonstrate that autistic females and their parents are acutely aware of subtle functional difficulties when compared to typically developing peers, a disparity reflected in the comparative data already discussed (Knickmeyer et al., 2008; Sedgewick et al., 2016; Sturrock et al., 2019b).

Therefore, despite a relatively subtle presentation of language and communication difficulties, autistic girls and boys without intellectual disability might be expected to experience a similar level of impact. Detail of that impact was provided in qualitative accounts (Sturrock et al., 2021) from 12 autistic children (6 girls, 6 boys). Daily living (participation and self-advocacy), social interrelations (social interactions and relationship-building) and emotional wellbeing (reactive and longer-term negative emotions and difficulties help-seeking) were all identified as areas of direct impact. Preliminary analysis of parental interviews (*n* = 12) seems to support these assumptions (Sturrock et al., in preparation). Supplementary Appendix 3 provides details of interviewee characteristics.

Thematic analysis found that difficulties with discourse, listening and word-finding were strongly associated with breakdown of conversations. These may contribute to results from recent empirical research, which suggests language difficulties will predict poorer social performance in autistic individuals (Levinson et al., 2020). Additionally, the associated effort incurred in managing these difficulties often resulted in avoidance or limitations to social participation. In child accounts, narrative difficulties were closely associated with limitations in explaining events, thoughts and ideas, and this in turn was related to difficulties with self-advocacy and social integration, as predicted in the literature (Dean et al., 2014; Sillar et al., 2014). Supplementary Appendix 4 shows a representative sample of quotes and themes.

These subtle difficulties experienced by autistic girls were also commonly associated with feelings of frustration, anxiety and negative sense of self-worth. The negative impact of communication difficulties on mental health are recognized in non-autistic populations (Levickis et al., 2018), but less well explored in the autism literature. This is an area of particular interest for future research due to the higher rates of associated mental health conditions in autistic individuals without intellectual disability (Leyfer et al., 2006).

The children interviewed not only described a negative emotional impact from communication difficulties, they (and their parents) also reported specific difficulties expressing emotional content in personal narratives. Recognition of emotion is thought to be limited in autistic individuals (Uljarevic and Hamilton, 2013) and this may be linked to underpinning difficulties with social cognition for the group (Löytömäki et al., 2020). However, recent research suggests that relative to autistic males, autistic females may be more inclined to comment on the emotions of others (Rieffe et al., 2021), they may have better skills in recalling emotional memory (Goddard et al., 2014), more advanced receptive and expressive use of vocabulary of emotion (Sturrock et al., 2019b) and improved narration of the internal states of others (Conlon et al., 2019; Kauschke et al., 2016). As emotional literacy is linked to better well-being (Eisenberg et al., 2005) through support-seeking and self-regulatory mechanisms, its relationship with sex/gender and communication difficulties is an important area of research interest.

## Discussion and Future Directions

This overview of the current literature strongly suggests that language and communication difficulties present differently for autistic females without intellectual disability, compared to autistic males with the same IQ and autism severity. This may contribute to poorer recognition and lower diagnostic rates of autism in this group. Areas of greatest sex/gender difference appear to exist in domains where meaning of structural language is mediated by social context; inference; language of emotion and internal state; and pragmatic behaviors (discourse and pragmatic features of narrative). See Table 1 for an overview of those findings.

**TABLE 1:**
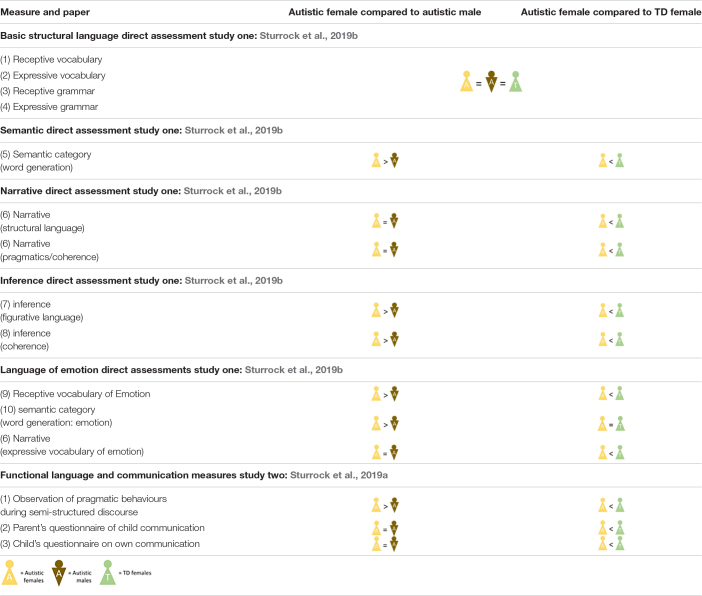
An overview of key findings showing a comparison between autistic females, autistic males, and TD females.

*Based on mean averages from a range of measures across two studies Sturrock et al., 2019a, b.*

Female advantages in pragmatic and semantic tasks may be linked to natural advantages in social motivation and attention, when compared to autistic males. This interaction should be explored and compared to the influence of higher-level linguistic skills.

Fewer studies provide sex/gender norms but where they do exist, autistic females appear to perform behind typically developing females on measures of pragmatics, semantics, and above sentence-level structural language. However, vocabulary and basic grammar (receptive and expressive) appear to be unaffected. Thus, the evidence reviewed suggests that measures of vocabulary and basic grammar cannot rule out higher-level language difficulty.

Further investigations are required to validate existing findings in a wider group, across different age ranges and with different IQ and autism severity. Other measures could also be explored with a particular focus on discourse analysis, spontaneously produced syntax and following instructions.

Perhaps surprisingly given these sex/gender differences in higher-level language abilities, questionnaire and interview data suggest that autistic females experience their language and communication difficulties similarly to autistic males, both in degree and type of impact reported. The parity of respondent accounts suggests that questionnaire and interview data may not be the best method for investigating sex/gender differences. The lack of observable differences when using these methods may reflect societal factors, with females and their parents naturally comparing their performance against the higher demands set by typically developing female groups. However, qualitative methods remain a critical tool for demonstrating the experience of the individual in both research and clinical domains.

Overall, then, it appears that the subtle language and communication difficulties outlined here may contribute to impact on functionality, social-interrelations and emotional well-being. These early findings should be consolidated with further empirical research. The relationship between subtle difficulties and emotional well-being is an area of particular concern due to the prevalence of mental health difficulties for this group.

### Clinical Implications

This paper supports the notion of a specific female autism phenotype and extends this to the domain of language and communication differences. Awareness of this presentation is essential for accurate identification and diagnosis of autistic females without intellectual disability.

The presentation of subtle language and communication difficulties, in particular above sentence-level language, pragmatics (inference and discourse) and semantics, should be assessed in clinical settings. This should include direct assessment, observations and facilitated self-report. Basic structural language measures of vocabulary and sentence-level grammar should not be used to rule out communication difficulties.

Results from appropriate assessments of need should be used to guide targeted interventions. This should include managing the negative impact of language and communication difficulties on functionality, social-interrelations and emotional well-being.

### Limitations

The literature in this area is sparse. It is also typified by smaller studies, and due to the wide range of measures, used overarching assumptions cannot be made with any certainty. In addition, many of the studies discussed are by necessity preliminary and exploratory. While these limitations mean that any conclusions drawn from the current paper must remain tentative, in itself this issue highlights an important point: linguistic profiles in the female autism phenotype are currently extremely poorly understood, and these gaps in our understanding may contribute to problems of mis- or under-diagnosis in this group. The current paper therefore highlights important avenues for future empirical work in this under-researched area.

## Data Availability Statement

The original contributions presented in the study are included in the article/Supplementary Material, further inquiries can be directed to the corresponding author/s.

## Ethics Statement

The studies involving human participants were reviewed and approved by South West—Central Bristol Research Ethics Committee (November 2015). Written informed consent to participate in this study was provided by the participants’ legal guardian/next of kin.

## Author Contributions

AS devised the research questions and was the lead researcher on the series of studies, which are discussed in this article. They were undertaken as part of her Ph.D., during which time she was supervised by JF and CA. All authors contributed to the development of methodology and data analysis across these studies. AS wrote this manuscript in consultation with this team of authors.

## Conflict of Interest

The authors declare that the research was conducted in the absence of any commercial or financial relationships that could be construed as a potential conflict of interest.

## Publisher’s Note

All claims expressed in this article are solely those of the authors and do not necessarily represent those of their affiliated organizations, or those of the publisher, the editors and the reviewers. Any product that may be evaluated in this article, or claim that may be made by its manufacturer, is not guaranteed or endorsed by the publisher.

## Footnotes

1 The term “sex/gender” is used to reflect the understanding that individuals’ identities are composed of hard to distinguish features of biological “sex” and socially constructed “gender.”
