# Supplementary material for: A Subtle Profile With a Significant Impact: Language and Communication Difficulties for Autistic Females Without Intellectual Disability
Source: Front Psychol. 2021 Aug 9;12:621742. doi: 10.3389/fpsyg.2021.621742 (PMC8380773; doi:10.3389/fpsyg.2021.621742)
Supplement: Supplementary file 1 [file Data_Sheet_1.docx]

Appendix 1: List of assessments, observation and report measures

| Appendix 1: Measures used in Sturrock, Yau, et al., (18) and Sturrock. Marsden, et al., (19) | | |
| --- | --- | --- |
| ***Item n^o^*** | ***Assessment name*** | ***Focus of assessment*** |
| Participant details | | |
| 1. | Wechsler Abbreviated Scale of Intelligence (WASI; Wechsler, 1999) | Performance IQ inclusion criteria |
| 2. | The Autism Spectrum Screening Questionnaire (ASSQ: Ehlers et al., 1999) | Autism inclusion criteria |
| Basic language direct assessments | | |
| 3. | British Picture Vocabulary Scale (BPVS-3) (Dunn et al., 1997) | Measure of basic structural language: receptive vocabulary |
| 4. | The Test of Word Knowledge (TOWK) expressive vocabulary subset (Wiig and Secord, 1992) | Measure of basic structural language: expressive vocabulary |
| 5. | The Test of Receptive Grammar – second edition (TROG-2) (Bishop, 2003) | Measure of basic structural language: receptive knowledge of grammar |
| 6. | The Clinical Evaluation of Language Fundamentals – fourth edition (CELF-4): Recalling Sentences task (Semel et al., 2006) | Measure of basic structural language:  expressive use of grammar |
| Above sentence-level language direct assessments | | |
| 7. | The Clinical Evaluation of Language Fundamentals – Fourth Edition (CELF-4): Understanding Spoken Paragraphs task (Semel et al., 2006) | Measures of higher level structural language: comprehension of above sentence level text |
| 8. | Expressive language sample in narrative  (type/token tally of complex syntactic markers) | Measures of higher level structural language: expressive above sentence level grammar |
| Pragmatic and semantic direct assessment | | |
| 9. | Expressive language sample in narrative | Measures of pragmatic and semantic skills: expressive use of coherence |
| 10. | Semantics: The Clinical Evaluation of language fundamentals – fourth edition (CELF-4): semantic word association tasks (Semel et al., 2006) | Measures of pragmatic and semantic skills: semantic word generation (animals, food, occupations) |
| 11. | Pragmatics: Figurative Language task (MacKay & Shaw, 2004) | Measures of pragmatic and semantic skills: pragmatic inference task |
| 12. | Pragmatics: Local Coherence task (Joliffe and Baron-Cohen, 1999) | Measures of pragmatic and semantic skills: pragmatic inference task |
| Language of emotion measures | | |
| 13. | Receptive Emotion Vocabulary (novel measure) | Measures of language of emotion: receptive vocabulary |
| 14. | Semantic word association task for language of emotion (novel measure) | Measures of language of emotion: semantic word generation |
| 15. | Expressive language sample in narrative  (tally of type/token) | Measures of language of emotion: spontaneous use in narrative |
| Functional communication observation and questionnaire measures | | |
| 16. | Pragmatic Rating Scale (PRS) (Landa, 1992, 2013): checklist for observable pragmatic communicative behaviours | Observation of pragmatic behaviours during semi-structured interviews |
| 17. | Child Communication Checklist – second edition (CCC-2) (Bishop, 2003): parent respondent | Parent rating of functional communication (social, pragmatic and structural language) |
| 18. | Communication Checklist - Self Report (CC-SR) (Bishop et al., 2009): child respondent | Child self-rating of functional communication (structural and pragmatic language and social communication) |
