# Supplementary material for: A Subtle Profile With a Significant Impact: Language and Communication Difficulties for Autistic Females Without Intellectual Disability
Source: Front Psychol. 2021 Aug 9;12:621742. doi: 10.3389/fpsyg.2021.621742 (PMC8380773; doi:10.3389/fpsyg.2021.621742)
Supplement: Supplementary file 2 [file Data_Sheet_2.docx]

Appendix 2:

Participant data for Sturrock, Yau et al (2019) and Sturrock, Marsden et al (2019)

| Appendix 2: Comparison by group (Autism/TD) and sex/gender (female/male) for chronological age (in months), PIQ scores (WISC), Autism severity (ASSQ) and basic language scores (BPVS, TOWK, CELF-4 Recalling Sentences) in Sturrock, Yau et al (18) and Sturrock, Marsden et al (19) | | | |
| --- | --- | --- | --- |
|  | Autism (*n* = 26)  Mean (*SD*) | TD (*n* = 26)  Mean (*SD*) | Sex/Gender overall  Mean (*SD*) |
| Age (in months) |  |  |  |
| Female | 124.46 (8.35) | 125.23 (6.98) | 124.85 (7.55) |
| Male | 118.31 (9.93) | 125.46 (7.88) | 121.88 (9.51) |
| Group overall | 121.39 (9.52) | 125.35 (7.29) | 123.37 (8.63) |
| PIQ (raw score) |  |  |  |
| Female | 107.69 (17.32) | 117.08 (14.95) | 112.38 (16.56) |
| Male | 106.46 (11.93) | 116.15 (13.10) | 111.31 (13.23) |
| Group overall | 107.08 (14.59) | 116.62 (13.78) | 111.85 (14.85) |
| Autism Severity  (ASSQ: max score 54) |  |  |  |
| Female | 32.83 (8.83) | 1.77 (2.77) | 16.68 (17.04) |
| Male | 34.42 (9.93) | 2.54 (3.76) | 17.84 (17.79) |
| Group overall | 33.63 (9.23) | 2.15 (3.26) | 17.26 (17.25) |
| BPVS-3: Receptive vocabulary  (max. score 132) |  |  |  |
| Female | 106.69  (16.34) | 104.38  (16.95) | 111.31  (5.63) |
| Male | 104.69  (16.65) | 107.15  (14.63) | 116.08  (7.70) |
| Group overall | 105.69  (16.19) |  | 113.69  (7.04) |
| TOWK: Expressive vocabulary  (max. score 29) | (n:25) |  |  |
| Female | 22.54  (3.46) | 13.00  (3.42) | 23.08  (2.84) |
| Male | 20.92  (4.65) | 12.69  (3.95) | 23.69  (3.33) |
| Group overall | 21.73  (4.09) |  | 23.40  (3.06) |
| CELF-4 Recalling Sentences subtest  (max score 95) |  |  |  |
| Female | 68.85  (10.13) | 10.69  (2.81) | 72.23  (12.71) |
| Male | 60.69  (13.76) | 9.08  (3.01) | 74.23  (10.83) |
| Group overall | 64.77  (12.55) |  | 73.23  (11.61) |

1. The groups were well-matched for chronological age measured in months: Group (*F*(1,48) = 2.924, *p* = .094, ŋ^2^ = .057); Sex/Gender (*F*(1, 48) = 1.634, *p* = .207, ŋ^2^ = .033); Group x Sex/Gender interaction (*F*(1,48) = 1.898, *p* = .175, ŋ^2^ = .038).
2. There was a small but significant main effect of Group on PIQ measured using the abbreviated Wechsler Intelligence Scale for Children (WISC) performance IQ subscale: (*F*(1,48) = 0.072, *p* =.021, ŋ^2^ = 0.105) with the TD group showing marginally higher PIQ (Mean = 116.62) than the Autism group (Mean = 107.08). There were no other significant effects on the PIQ measure (Sex/Gender: *F*(1,48) = 0.072, *p* = .790, ŋ^2^ = .001; Group x Sex/Gender interaction: *F*(1,48) = 0.001, *p* = .970, ŋ^2^ = .000). Between group analyses were corrected for PIQ.
3. There was a significant effect of Group on Autism severity ratings measured using the Autism Spectrum Screening Questionnaire (ASSQ): (*F*(1,46) = 257.966, *p* = - .001, ŋ^2^ = 0.849) with TDs showing lower scores, i.e. fewer difficulties, on the ASSQ (Mean = 2.15) than the Autism group (Mean = 33.36). There were no other significant effects on the ASSQ scores (Sex/Gender: *F*(1,46) = 0.360, *p* = .551, ŋ^2^ = .008; Group x Sex/Gender interaction: *F*(1,46) = 0.043, *p* = .836, ŋ^2^ = .043). As this was an expected group difference and did not directly impact on analysis of sex/gender difference. It was not introduced as a covariate in subsequent analysis, because severity of autistic symptomatology (in terms of language and communication) was a key factor under investigation.
4. There was a small but significant main effect of Group using the British Picture Vocabulary Scale (BPVS) receptive vocabulary measure: (*F*(1, 48) = 5.241, *p* = .026, ŋ^2^ = .098) as the TD participants (mean = 113.69) outperformed the Autism participants (mean = 105.69). This was no longer significant when controlling for PIQ (*F*(1, 47) = 1.892, *p* = .176, ŋ^2^ = .039). There was no significant effect of Sex/Gender (*F*(1, 48) = 0.157, *p* = .694, ŋ^2^ = .003) and no significant Group x Sex/Gender interaction (*F*(1, 48) = 0.938, *p* = .338, ŋ^2^ = .019).
5. There were no significant effects on the Expressive vocabulary subtest (TOWK), expressive vocabulary measure: Group: *F*(1, 47) = 2.634, *p* = .111, ŋ^2^ = .053; Sex/Gender: *F*(1, 47) = 0.243, *p* = .624, ŋ^2^ = .005; Group x Sex/Gender: *F*(1, 47) = 1.187, *p* = .282, ŋ^2^ = .025
6. There was a small and significant main effect of Group using the Clinical Evaluation of Linguistic Fundamentals (CELF) Recalling Sentences subtest: (*F*(1, 48) = 6.525, *p* = .014, ŋ^2^ = .120) as the TD participants had more correct items (mean = 73.23) than the Autism participants (mean = 64.77). This was no longer significant after controlling for PIQ (*F*(1, 47) = 2.89, *p* = .096, ŋ^2^ = .058). Neither the main effect of Sex/Gender (*F*(1, 48) = 0.863, *p* = .358, ŋ^2^ = .018) or the Group x Sex/Gender interaction was significant (*F*(1, 48) = 2.349, *p* = .132, ŋ^2^ = .047).
