# Supplementary material for: A Subtle Profile With a Significant Impact: Language and Communication Difficulties for Autistic Females Without Intellectual Disability
Source: Front Psychol. 2021 Aug 9;12:621742. doi: 10.3389/fpsyg.2021.621742 (PMC8380773; doi:10.3389/fpsyg.2021.621742)
Supplement: Supplementary file 3 [file Data_Sheet_3.docx]

Appendix 3

| Appendix 3: Demographics of children and parent respondents to interviews (20, 21) focussing on subtle language and communication profiles of autistic children without intellectual impairment and its impact | | | | | |  |
| --- | --- | --- | --- | --- | --- | --- |
| Parent interviewee | Child interviewee* | Child’s Sex/Gender | Child’s Age in Years | Child’s Diagnosis | Recruitment channel | |
| Mother | Gemma | Female | 13 | Autism | **Charity network | |
| Mother | Emily | Female | 11 | Autism/anxiety | Autistic girls social media site | |
| Mother | Esther | Female | 12 | Autism/ adhd | **NHS | |
| Mother | Alisa | Female | 9 | Autism/spd | PPIE network | |
| Mother | Molly | Female | 14 | Autism/ dyspraxia | **Charity network | |
| Mother | Lottie | Female | 9 | Autism | PPIE network | |
| Mother | Liam | Male | 12 | Autism | **NHS | |
| Mother | Jacob | Male | 11 | Autism/adhd | **Charity network | |
| Mother | Andrew | Male | 12 | Autism/adhd | **NHS | |
| Mother | Fletcher | Male | 11 | Autism | **NHS | |
| Mother | Lucas | Male | 12 | Autism/ dyspraxia | **Charity network | |
| Mother | Oscar | Male | 13 | Autism | **Charity network | |
| *Pseudonyms used throughout **Recruited originally study (Sturrock et al, 2019)  Acronyms: Attention Deficit and Hyperactivity Disorder (ADHD) Sensory Processing Disorder (SPD) | | | | | |  |
