# Supplementary material for: A Subtle Profile With a Significant Impact: Language and Communication Difficulties for Autistic Females Without Intellectual Disability
Source: Front Psychol. 2021 Aug 9;12:621742. doi: 10.3389/fpsyg.2021.621742 (PMC8380773; doi:10.3389/fpsyg.2021.621742)
Supplement: Supplementary file 4 [file Data_Sheet_4.docx]

| Appendix 4: Sample quotes from child (20) and parent (21) interviews on the impact of subtle language and communication difficulties for autistic females (and males) | | |
| --- | --- | --- |
| **Listening & comprehension difficulties; impact on daily function and education** | **Child accounts:** | ‘in school when they’re telling us what to do I just drift off and I don’t know what we’re doing’ |
|  | **Parent accounts:** | ‘what I’ve learnt is to just ask her to do one thing at a time. If I give her a list (...) She’ll just say ‘I don’t know what I have to do’ |
| **Narrative difficulties and impact on explaining personal events** | **Child accounts:** | ‘sometimes I’ll be ok, but go into loads of detail, or I’ll barely go into any detail, to try to avoid going into loads of detail’ |
|  | **Parent accounts:** | ‘everything’s intertwined. Then she’ll go onto something else without finishing the first thing’ |
| **Additional difficulties explaining events with emotional content** | **Child accounts:** | ‘usually I’ll convey a negative emotion, of being sad or worried, but I’ll shut down and won’t be able to express a negative emotion. So they’ll know it’s a negative emotion, but not which one’ |
|  | **Parent accounts:** | ‘if it’s emotional topics (...) she would clam up really quickly; I don’t think she would engage in a conversation |
| **Interaction between subtle language & communication difficulties & creating & maintaining social relationships** | **Child account:** | ‘I need to know them really well before I can talk to them, and they need to talk to me first’ |
|  | **Parent account:** | ‘that’s maybe how the friendships could be stronger if she did more chatting to them’ |
| **Interaction between language & communication difficulties & negative emotional well-being** | **Child account:** | ‘if I’m sad and I can’t express my sadness I usually just become more sad, so it makes the emotion worse (...) I might be angry with myself that I can’t convey the emotion, or angry at other people because they’re not letting me convey the emotion’ |
|  | **Parent account:** | ‘I think she gets a bit too worked up to explain things thoroughly sometimes, then it causes problems’ |
